# Ten-Year Trend in the Potentially Inappropriate Prescribing of Renally-Dependent Medicines in Australian General Practice Patients with Dementia

**DOI:** 10.3390/jcm14134734

**Published:** 2025-07-04

**Authors:** Saad Alhumaid, Woldesellassie M. Bezabhe, Mackenzie Williams, Gregory M. Peterson

**Affiliations:** School of Pharmacy and Pharmacology, University of Tasmania, Hobart 7006, Australia; woldesellassie.bezabhe@utas.edu.au (W.M.B.); mackenzie.williams@utas.edu.au (M.W.); g.peterson@utas.edu.au (G.M.P.)

**Keywords:** dementia, potentially inappropriate prescribing, prevalence, kidney, dose

## Abstract

**Background:** There is limited published evidence on the prevalence of potentially inappropriate prescribing of medicines in relation to kidney function in older Australians, particularly those with dementia. **Objectives:** To examine the prevalence, temporal trends and factors associated with potentially inappropriate prescribing of renally-dependent medicines in patients with dementia, using Australian general practice data. **Methods:** This comparative study was reported in accordance with the STROBE guidelines for cohort studies. Retrospective analyses of the National Prescribing Service (NPS) MedicineInsight dataset were performed to determine the proportion of patients aged ≥ 65 years with a recorded diagnosis of dementia, along with matched controls, who had potentially inappropriate prescribing based on their estimated glomerular filtration rate (eGFR) during the study period (2011–2020). Each patient was included only once throughout the study. Potentially inappropriate prescribing was evaluated for 33 commonly used medicines, using the Cockcroft-Gault equation for estimated creatinine clearance or eGFR, in accordance with the guidelines from the Australian Medicines Handbook (AMH). Each patient’s medicines were included if they were prescribed within 180 days after the most recent recorded lowest eGFR value for the patient. Medicines having prescribed doses exceeding those recommended for an individual’s renal function were classified as ‘inappropriate dosage’, while those whose use was advised against were labelled ‘contraindicated’. Both categories were regarded as inappropriate prescriptions. Descriptive statistics were used to summarise patient characteristics and medication use. Temporal trends were displayed in graphs, with statistical significance determined using the Cochran-Armitage test. Binary logistic regression models were used to examine the associations between sociodemographic and clinical factors and the prescribing of medicines inconsistent with AMH guidelines. **Results:** The unmatched cohorts included 33,101 patients, comprising 4092 with dementia and 29,009 without. Among them, 58.4% were female, and the overall median age was 82 years [interquartile range (IQR): 77–87]. After propensity score matching, there were 4041 patients with dementia and 8031 without dementia. Over the study period, potentially inappropriate prescribing increased slightly, but insignificantly, in both groups of patients; the prevalence of inappropriate use of at least one of the 33 drugs of interest rose from 6.5% (95% CI 4.5–9.1%) in 2011 to 8.9% (95% CI 6.0–12.7%; *p* for trend: 0.966) in 2020 in the dementia group, and 9.2% (95% CI 8.0–10.5%) to 11.1% (95% CI 10.3–12.0%; *p* for trend: 0.224) in the matched controls. Over the ten-year period, approximately 9.3% (377) of patients with dementia in the matched cohort received at least one potentially inappropriate prescription. Among these, 154 (40.8%) were for contraindicated medicines, and 223 (59.1%) were for inappropriate doses based on renal function. Among patients with dementia in the matched cohort, fenofibrate, nitrofurantoin, and moxonidine were the most frequently prescribed medicines at doses inconsistent with AMH guidelines. In the unmatched dementia cohort, potentially inappropriate prescribing was not significantly associated with demographic characteristics or most comorbidities; however, it occurred more frequently in patients with an eGFR below 30 mL/min/1.73 m^2^ or those with concomitant diabetes. **Conclusions:** Positively, the prevalence of potentially inappropriate prescribing of renally-dependent medicines in primary care patients with dementia in Australia was similar to their matched controls. However, there was room for improvement in the prescribing of these drugs in both patients with and without dementia.

## 1. Introduction

Chronic kidney disease (CKD) is a common condition that frequently goes undiagnosed, especially among older adults [1]. This lack of recognition increases the risk of inappropriate medication use [2]. A key concern is the prescribing of renally-dependent medicines—those requiring dose adjustments or avoidance in cases of impaired kidney function—which remains a challenging issue in clinical practice [3]. Despite clinical guidelines, such medicines are often prescribed inappropriately to older patients with CKD [4]. In patients with renal impairment, inappropriate prescribing has been associated with significantly higher rates of adverse drug events, emergency department visits, prolonged hospitalisation, and, in severe cases, increased mortality [5,6,7,8].

Dementia is another condition that becomes increasingly prevalent with advancing age [9]. As the population ages, the number of individuals affected by dementia continues to rise, bringing serious challenges to clinical care and medication management [10]. Several factors contribute to the elevated potentially inappropriate medication risk in patients with dementia compared to those without, including cognitive impairment, poor medication adherence, polypharmacy, multiple comorbidities, and a higher likelihood of drug–drug interactions [11,12]. Communication barriers and reduced clinical monitoring may result in less individualized and suboptimal care [13,14]. Compared with non-dementia, those with dementia are also more likely to have additional chronic illnesses—such as hypertension and diabetes—further increasing their risk of polypharmacy [15]. Older adults with dementia are more likely to be prescribed five or more concurrent medicines and are at an increased risk of experiencing drug–drug interactions [16,17]. In advanced stages of dementia, clinicians may intentionally deprioritize renal dosing considerations in favour of a comfort-oriented approach, placing emphasis on symptom relief rather than long-term risk mitigation [18,19].

The coexistence of dementia and CKD presents a uniquely complex landscape for prescribing decisions [20]. Research consistently shows that individuals with dementia are more likely to receive potentially inappropriate medicines [11,12,21], a risk further heightened by impaired renal function [22]. Previous literature reviews have explored the prevalence and outcomes of potentially inappropriate prescribing in older adults with dementia, often employing screening tools such as the STOPP or Beers criteria [11,12,21]. However, these studies have not specifically considered CKD as a potential risk modifier for inappropriate prescribing within this population [11,12,21]. Our recent systematic review identified a notable gap in the literature surrounding the dosing appropriateness of renally-cleared medicines in older adults with dementia or cognitive impairment [23]. Based on the limited published data, we found that inappropriate drug dosing in such patients occurs frequently [23]. These considerations highlight the critical importance of careful medication management in older adults with co-existing dementia and renal impairment.

To date, rates of potentially inappropriate prescribing of renally-dependent medicines in patients with diabetes, hypertension, cardiovascular disease and atrial fibrillation have been well documented [24,25,26,27], but the frequency of potentially inappropriate prescribing and potential differences in older people with dementia, compared to those without dementia, have not been characterised. We aimed to examine the prevalence and temporal trends of potentially inappropriate prescribing based on kidney function, as well as the factors associated with potentially inappropriate prescribing, focusing on older Australians with dementia.

## 2. Methods

### 2.1. Design and Data Source

This comparative study was reported in accordance with the STROBE guidelines for cohort studies (Supplementary File S1) [28]. A retrospective analysis was undertaken using primary care data obtained from the National Prescribing Service (NPS) MedicineWise’s dataset (MedicineInsight). The data spanned from 1 January 2011 to 31 December 2020. MedicineInsight contains anonymised patient demographics, clinical records, prescriptions, and pathology results from general practices nationwide across Australia. Demographic characteristics of the patients are largely representative of the broader Australian population in terms of age, gender and socioeconomic status [29]. Since the closure of NPS MedicineWise, stewardship of MedicineInsight has transitioned to the Australian Commission on Safety and Quality in Health Care. Further details on the dataset have been documented previously [30] and it has served as a data source for multiple published studies [1,24,31].

### 2.2. Study Population and Inclusion Criteria

For this study, we obtained records of patients aged ≥ 65 years, with and without a documented dementia diagnosis, from MedicineInsight. Patients with at least one recorded renal function test result, specifically the estimated glomerular filtration rate (eGFR), were selected for further analysis. Patients with at least three recorded general practice visits in the two years preceding the index date, defined as the date of the individual’s most recently recorded lowest eGFR value during the study period (2011–2020), were initially included. Each patient was included only once throughout the study. Patients with dementia were required to have at least one year of data following their recorded dementia diagnosis, and those whose diagnosis date was not recorded or occurred after 31 December 2019 were excluded.

### 2.3. Study Outcomes, Study Covariates and Statistical Analysis

To identify potentially inappropriate prescribing, recommendations in the Australian Medicines Handbook (AMH) were applied according to the renal function of each patient [32]. We focused on a list of 33 drugs that are commonly prescribed in general practice and are recommended to be avoided or used with dosage adjustment in patients with renal impairment. The drug list was adapted from an earlier publication [25]. Only medicines that had clear dosage recommendations for renal impairment in the AMH were included (Supplementary Table S1). The included patients, both with and without dementia, had been prescribed at least one of the 33 drugs of interest during the study period. Each patient’s medicines were included for analysis if prescribed within 180 days after the index date’s eGFR test, with the latest prescription considered for the same medicine to allow more time for the prescriber to alter the therapy following renal function testing.

Medicines that were available in combination products were assessed for potentially inappropriate prescribing individually (e.g., metformin and sitagliptin). For each drug, prescriptions were marked as ‘appropriate dosage’ when the prescribed dose was in conformity with the adjustment specified in the AMH with respect to the patient’s renal function [32]. Medicines were considered as having an ‘inappropriate dosage’ when the prescribed dose exceeded the maximum recommended for the patient’s renal function. Medicines were considered as ‘contraindicated’ if the AMH recommended avoiding their use in renal impairment on the basis of the patient’s individual renal function. Both ‘inappropriate dosage’ and ‘contraindicated’ prescriptions were treated as inappropriate prescriptions. It is important to note that the AMH recommendations use estimated creatinine clearance (CrCl), not eGFR, when providing guidance about prescribing for people with renal impairment. Our previous work showed that agreement between the eGFR and CrCl was excellent using the AMH, with 97% of the medicines rated as appropriately dosed by eGFR being also rated as appropriate using the Cockroft-Gault CrCl equation [25]. Prescriptions with no dosage or frequency recorded, or dosage documented as “take as directed” (“mdu”) or “immediate”, were coded as missing and excluded from the analysis.

Descriptive statistics were used to summarize sociodemographic and clinical characteristics of patients with and without dementia in both unmatched and matched cohorts, reported as medians with interquartile ranges (IQRs), percentages, or proportions. In the matched cohorts, we examined trends in the inappropriate use of at least one of the 33 drugs of interest among patients with dementia versus those without, spanning the period from 2011 to 2020. We first constructed a sample of patients without a documented diagnosis of dementia (controls) by matching them to dementia cases across the entire study period. Matching variables included age, sex, rurality, general practice location (by Australian state or territory location), socioeconomic status, index year, frequency of general practitioner (GP) visits in the preceding two years, and the presence of specific medical conditions. These comorbidities included heart failure, hypertension, stroke, atrial flutter, anxiety, arthritis, asthma, diabetes, deep vein thrombosis, depression, cancer, coronary artery disease, chronic liver disease, chronic obstructive pulmonary disease, atrial fibrillation, substance abuse, osteoporosis, chronic pain, and schizophrenia. Comorbid conditions were identified using algorithms that analysed both coded data and free-text entries within the ‘encounter_reason’, ‘prescription_reason’, and ‘diagnosis_reason’ fields [33]. The Index of Relative Socio-Economic Advantage and Disadvantage (IRSAD), developed by the Australian Bureau of Statistics (ABS), provides a summary measure of socioeconomic status across geographic areas based on census-derived variables such as income, education, employment, occupation, and housing. Higher IRSAD scores reflect more socioeconomically advantaged communities [34]. This index contributes to the ABS’s broader Socio-Economic Indexes for Areas (SEIFA), which classify areas into quintiles ranging from 1 (most disadvantaged) to 5 (most advantaged) [34]. Geographic remoteness was assessed using the Accessibility/Remoteness Index of Australia (ARIA), categorised into five levels: major cities (ARIA 0–0.20), inner regional (0.21–2.40), outer regional (2.41–5.92), remote (5.93–10.53), and very remote (10.54–15) [35]. Due to the relatively small sample sizes in the outer regional, remote and very remote areas, these were grouped together into a single category. Thus, rurality was condensed into three categories: major cities, inner regional, and outer regional/remote/very remote. Additionally, the Australian Capital Territory was grouped with New South Wales, and the Northern Territory with South Australia, resulting in six regional categories: New South Wales/Australian Capital Territory, Victoria, Queensland, Western Australia, Tasmania, and South Australia/Northern Territory. Propensity score matching followed a 2:1 ratio (controls to cases) and was performed without replacement, in descending order of match quality [36,37]. Matching employed a calliper of 0.02 on the logit scale of the propensity score [38,39], and any absolute standardised difference equal to or exceeding 0.10 was considered indicative of a meaningful imbalance.

In the matched cohorts, the use of the 33 drugs of interest was quantified by drug class and active ingredient. The count and proportion of patients, both with and without dementia, who had inappropriate use of these drugs were summarised and stratified by eGFR categories (≥30 mL/min/1.73 m^2^ and <30 mL/min/1.73 m^2^). For each year, we estimated the proportion of patients—with and without dementia—who were inappropriately prescribed at least one renally-dependent medicine. These estimates were accompanied by 95% confidence intervals (CIs), calculated using the exact binomial approach. Temporal trends were shown in graphs, and the Cochran-Armitage trend test was applied to evaluate statistical significance (accompanied by *p*-values from Pearson’s chi-square analyses) [40]. To assess differences in proportions between individuals with dementia and their matched counterparts regarding inappropriate use of at least one of the 33 targeted medicines, a two-sample z-test was employed [41]. Additionally, a sensitivity analysis was performed to explore prescribing trends throughout the study period in both dementia and control groups without applying matching criteria.

We analysed sociodemographic and clinical characteristics of unmatched dementia patients using data from 2011 to 2020 to identify factors associated with the potentially inappropriate prescribing of at least one of the 33 drugs of interest. After checking multicollinearity, we performed a multivariate logistic regression. Peripheral vascular disease, initially part of the model, was excluded due to its multicollinearity with coronary heart disease. Moreover, variables such as general practice site (Australian state/territory location), number of GP visits in the past two years, Aboriginal and Torres Strait Islander (ATSI) status, number of renally-dependent drugs prescribed, and specific medical comorbidities (heart failure, atrial flutter, anxiety, arthritis, asthma, deep vein thrombosis, depression, coronary heart disease, chronic liver disease, substance abuse, osteoporosis, pain, and schizophrenia) were excluded from the model due to insignificance in univariate analyses. The dependent variable was the presence of inappropriate medication use, while the final independent variables included age, sex, index year, rurality, socioeconomic status, eGFR category (≥30 mL/min/1.73 m^2^ and <30 mL/min/1.73 m^2^), and the presence of specific medical comorbidities (hypertension, stroke, diabetes, cancer, chronic obstructive pulmonary disease, and atrial fibrillation). A backwards selection procedure was implemented to determine the final regression model.

Data manipulation was carried out using SAS version 9.4 (SAS Institute Inc., Cary, NC, USA). Microsoft Excel 2019 (Microsoft Corp., Redmond, WA, USA) and IBM SPSS Statistics 29.0 (IBM Corp., Armonk, NY, USA) were employed for data cleaning and statistical analysis. Statistical significance was determined at a threshold of *p* <0.05.

### 2.4. Ethical Considerations

This study received ethical approval from the Human Research Ethics Committee at the University of Tasmania (HREC Project ID 20006), along with approval from the MedicineInsight independent Data Governance Committee. As the data were anonymised, individual patient consent was not required.

## 3. Results

**Figure 1:**
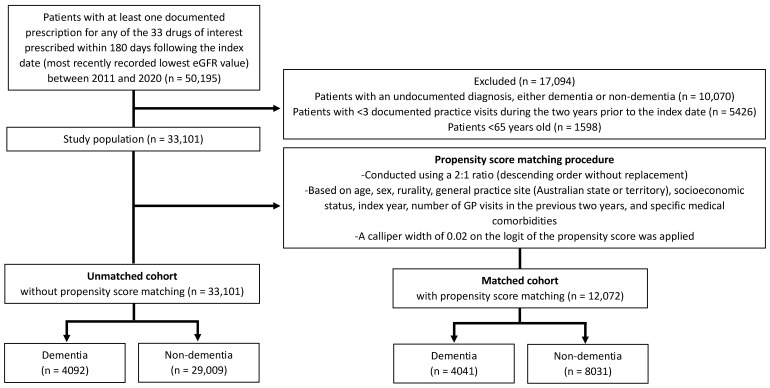
Flow chart of patient inclusion. GP, general practitioner; eGFR, estimated glomerular filtration.

The unmatched cohorts included 33,101 patients, comprising 4092 with dementia and 29,009 without. Among them, 58.4% were female, and the overall median age was 82 years (IQR: 77–87). Patient inclusion is shown in Figure 1. The characteristics of patients in the dementia and non-dementia groups, both before and after propensity score matching, are detailed in Table 1. Through propensity score matching, we successfully paired 12,072 patients, including 4041 with dementia and 8031 without dementia.

**Figure 2:**
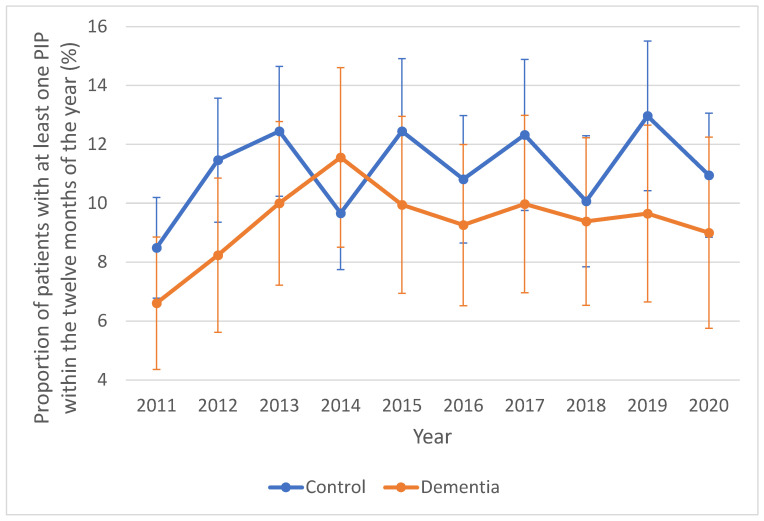
Trends in potentially inappropriate prescribing of 33 renally-dependent drugs in primary care patients with dementia and their matched controls. Error bars indicate 95% confidence intervals. PIP, potentially inappropriate prescription.

Of the 33 drugs examined, rosuvastatin was the most commonly prescribed for both included patients with dementia (17.7%) and their matched controls (17.8%). This was followed by digoxin (11% and 11.9% for patients with dementia and matched controls, respectively), metformin (9.2% and 8.1%), and pregabalin (8.6% and 11%) (Table 2). The key changes in potentially inappropriate prescribing of the 33 drugs over time are shown in Figure 2 and Supplementary Figures S1 and S2. It slightly increased, but insignificantly, in both groups, from 6.5% (95% CI: 4.5–9.1%) in 2011 to 8.9% (95% CI: 6.0–12.7%) in 2020 (*p*-value for trend: 0.966) in the dementia group, and 9.2% (95% CI: 8.0–10.5%) in 2011 to 11.1% (95% CI: 10.3–12.0%) in 2020 (*p*-value for trend: 0.224) in the matched controls (Figure 2). The trend in potentially inappropriate prescribing calculated using unmatched dementia and control groups (sensitivity analysis) was similar to that obtained from the matched groups (Supplementary Figure S1).

Across all study years, the prevalence of potentially inappropriate prescribing in the dementia group was slightly lower than that in the matched controls, except in 2014. However, the differences were not statistically significant. Over the ten-year period, 377 patients with dementia (9.3%) received prescriptions for potentially inappropriate dosages, compared to 885 patients (11%) in the matched control group. Among the 377 patients with dementia, 154 (40.8%) received contraindicated medicines, and 223 (59.1%) were prescribed doses inappropriate for their kidney function. In comparison, 404 patients (45.6%) in the matched control group received contraindicated medicines, and 481 patients (54.4%) were given inappropriate doses based on kidney function. Among patients with dementia in the matched cohort, fenofibrate (33/49, 67%), nitrofurantoin (40/64, 62%), and moxonidine (15/47, 31%) had the highest proportions of doses that were inconsistent with AMH guidelines for renal impairment. In the matched control group, the highest proportions of such inconsistencies were noted for fenofibrate (67/105, 63.8%), nitrofurantoin (72/108, 66%), nizatidine (35/72, 48%), and famotidine (13/32, 40%) (Table 2). Across both matched cohorts, patients with or without dementia who had more advanced CKD (eGFR <30 mL/min/1.73 m^2^) showed higher rates of potentially inappropriate prescribing compared to those in earlier CKD stages (eGFR ≥30 mL/min/1.73 m^2^). Among patients with dementia and advanced CKD, the drug classes most frequently prescribed at doses inconsistent with AMH guidelines included aldosterone antagonists (spironolactone), alpha-2/imidazoline receptor agonists (moxonidine), antibiotics (nitrofurantoin), and fibrates—all of which showed 100% inappropriateness based on renal function. Similarly, in the matched control group with advanced CKD, these drug classes, along with H_2_ antagonists, also showed 100% inconsistency with AMH guidelines (Supplementary Table S2). Sub-analyses based on eGFR severity (≥30 mL/min/1.73 m^2^ and <30 mL/min/1.73 m^2^) in the matched cohorts showed no statistically significant differences in the rates of potentially inappropriate prescribing between patients with and without dementia (Supplementary Figure S2).

From 2011 to 2020, sub-analyses across both matched cohorts were performed by age groups, gender, rurality, SEIFA quintiles, general practice site (Australian state/territory location), ATSI status, and frequency of GP visits in the preceding two years. These analyses revealed no statistically significant differences in the rates of potentially inappropriate prescribing between patients with and without dementia across the various sub-groups.

In the unmatched dementia cohort, variables potentially associated with at least one potentially inappropriate prescribing in patients with dementia from 2011–2020 were analysed through binary logistic regression analyses, and the results are shown in Table 3. Adjusted regression analysis revealed that patients with dementia were more likely to receive at least one inappropriate prescription if they belonged to the eGFR category of <30 mL/min/1.73 m^2^, or if they had diabetes. Conversely, patients with atrial fibrillation showed a significantly lower rate of potentially inappropriate prescribing compared to those without this condition (Table 3).

## 4. Discussion

This study examined the prescribing trends of 33 renally-dependent medicines in the primary care setting for older adults with dementia. Notably, the prevalence of potentially inappropriate prescribing—defined as prescribing doses that exceed recommendations or involve contraindicated medicines based on renal function—did not differ significantly between patients with and without dementia. Approximately 9.3% of patients with dementia received at least one potentially inappropriate prescription, being inconsistent with AMH guidelines for renal impairment.

The drug classes most linked to potentially inappropriate prescribing were aldosterone antagonists (spironolactone), alpha-2/imidazoline receptor agonists (moxonidine), antibiotics (nitrofurantoin), and fibrates (fenofibrate). The medicines identified as potentially inappropriate for patients with dementia were largely consistent with previous studies on prescribing practices for individuals with renal impairment and dementia [22,42,43]. Antihypertensives and lipid-lowering agents are frequently prescribed for individuals with dementia [44], increasing the risk of polypharmacy and potentially inappropriate prescribing [21]. This is expected due to the common coexistence of hypertension and cardiovascular diseases with CKD [45]. Around 75% of patients were treated for hypertension, and 20% for cardiovascular conditions, including coronary heart disease, heart failure, and stroke. Many of these medicines are renally excreted, requiring frequent adjustments and heightening the risk of inappropriate prescribing [46,47]. Fenofibrate was the most frequently prescribed medicine at doses exceeding AMH guidelines (67% of cases). This aligns with findings from studies in France and Australia, which reported high rates of inappropriate fenofibrate prescribing in community-dwelling elderly individuals with impaired kidney function [26,48]. Fenofibrate is contraindicated for patients with an eGFR below 30 mL/min, yet it is sometimes prescribed for patients with an eGFR as low as 20 mL/min in clinical practice [49]. This may be due to patient-specific assessments, where cardiovascular benefits are deemed to outweigh the risks [50]. Severe hypertriglyceridemia and varying interpretations of guidelines among GPs also contribute to this discrepancy [49,51].

Of concern was the high proportion (62.5%) of nitrofurantoin prescriptions exceeding guideline-recommended doses in patients with dementia. A Canadian study reported that 100% of older adults with CKD Stages 4 and 5 in primary care received nitrofurantoin at inappropriate doses [52]. Increased resistance to sulfamethoxazole/trimethoprim and fluoroquinolones may explain the growing use of nitrofurantoin among the elderly [53]. However, decreased renal function, multiple comorbidities, and increased drug therapy in elderly patients necessitate careful consideration [54]. Reassuringly, the dosing of anti-dementia drugs, memantine and galantamine, was 100% appropriate in relation to renal function.

Prescriptions with potentially inappropriate prescribing showed no significant correlation with demographics or most comorbidities in patients with dementia, but were more frequent in those with an eGFR below 30 mL/min/1.73 m^2^ or diabetes. Previous research on patients with advanced CKD has shown mixed results. While some studies indicate that enhanced monitoring and regular nephrology consultations reduced the incidence of potentially inappropriate prescribing [55,56], others have reported a higher frequency of prescribing-related issues [57,58,59]. Diabetes, a well-established risk factor for inappropriate prescribing in patients with CKD [26], frequently necessitates the use of medicines like biguanides (metformin) and DPP-4 inhibitors (sitagliptin, alogliptin and saxagliptin) [60]. These drugs often require dose adjustments in renal impairment [60]. Over 30% of our patients had diabetes, emphasising its influence on prescribed medicines, including frequent metformin use. Australian guidelines recommend annual assessments for renal impairment in individuals with diabetes [61], explaining more frequent eGFR monitoring by GPs in dementia patients with coexisting diabetes [62]. The lower rates of potentially inappropriate prescribing in patients with atrial fibrillation could be attributed to the structured guidelines and protocols for managing atrial fibrillation [63,64]. These guidelines emphasise the use of specific medicines and renally-adjusted doses [64,65]. GPs may also exercise greater caution when prescribing for patients with atrial fibrillation due to bleeding risks, resulting in more appropriate prescribing practices or even potentially under-dosing [66,67,68].

Studies suggest that a range of interventions—including computerised, manual, and educational approaches—can significantly reduce inappropriate prescribing in patients with CKD and mitigate associated adverse outcomes, with the most substantial benefits observed in pharmacist- or clinician-led initiatives [69]. These interventions should focus on renally-dependent medicines commonly prescribed to older adults with both dementia and renal impairment. Given the well-established association between potentially inappropriate prescribing of renally-dependent medicines and increased risks of adverse drug events, unplanned emergency department visits, prolonged hospitalisation, and mortality [5,6,7,8], healthcare professionals should prioritise addressing this problem in this vulnerable population.

### Strengths and Limitations

To the best of our knowledge, no other study has looked specifically at trends of potentially inappropriate prescribing of renally-dependent medicines over time in primary care patients with dementia. Our study was based on a large, community-based nationally representative data source. It examined trends of potentially inappropriate prescribing over a 10-year period. Propensity score matching was used to adjust for patients’ demographic and clinical characteristics. We also characterised whether prescriptions were consistent with guidelines utilising eGFR results which would have been available to the GP preceding the issuing of a prescription. We acknowledge several limitations in our study. Firstly, we did not investigate adverse clinical outcomes associated with potentially inappropriate prescribing. Secondly, the prescribing of seemingly inappropriate doses may sometimes be clinically justified in individual cases, where the benefits outweigh the associated risks or when no safer alternatives are available. Thirdly, the sample sizes for some of the individual drugs examined were relatively small. Lastly, the MedicineInsight dataset comprises unique patient records rather than distinct individuals, introducing a small possibility of duplicate entries for patients attending multiple practices.

## 5. Conclusions

In summary, the prevalence of potentially inappropriate prescribing based on renal function was not significantly different in patients with dementia compared to their matched controls. Approximately 9.3% of patients with dementia received at least one potentially inappropriate prescription that was inconsistent with the AMH guidelines for renal impairment. Such prescriptions were more common among patients with an eGFR below 30 mL/min/1.73 m^2^ or those with diabetes.

## Figures and Tables

**Table 1:** Baseline characteristics of dementia vs. non-dementia patients (2011–2020), pre- and post-propensity score matching.

Characteristics	Before Matching			Propensity-Score Matched		
	Dementia (*n* = 4092)	Non-Dementia (*n* = 29,009)	Standardized Differences *	Dementia (*n* = 4041)	Non-Dementia (*n* = 8031)	Standardized Differences *
Age (years), median (IQR)	81 (76–86)	82 (77–87)	0.167	81 (76–86)	81 (76–86)	0.005
Age group (years)			0.159			0.013
65–74	855 (20.9)	4555 (15.7)		840 (20.8)	1620 (20.2)	
75–84	1919 (46.9)	13,344 (46)		1895 (46.9)	3789 (47.2)	
≥85	1318 (32.2)	11,110 (38.3)		1306 (32.3)	2622 (32.6)	
Sex-female (%)	2355 (57.6)	16,982 (58.4)	0.016	2329 (57.6)	4641 (57.8)	0.003
Index year (%)			0.356			−0.014
2011–2012	913 (22.3)	4312 (14.9)		894 (22.1)	1906 (23.7)	
2013–2014	886 (21.7)	4755 (16.4)		874 (21.6)	1781 (22.2)	
2015–2016	825 (20.2)	4966 (17.1)		814 (20.1)	1486 (18.5)	
2017–2018	793 (19.4)	5974 (20.6)		786 (19.5)	1338 (16.7)	
2019–2020	675 (16.5)	9002 (31)		673 (16.7)	1520 (18.9)	
Aboriginal and Torres Strait Islander (%)			0.041			−0.017
Yes	31 (0.8)	191 (0.7)		31 (0.8)	48 (0.6)	
No	3284 (80.3)	23,807 (82.1)		3243 (80.3)	6418 (79.9)	
Unknown	777 (19)	5011 (17.3)		767 (19)	1565 (19.5)	
Rurality (%)			−0.091			−0.015
Outer regional, remote and very remote	2155 (52.7)	16,342 (56.3)		2150 (53.2)	4245 (52.9)	
Inner regional	1314 (32.1)	9170 (31.6)		1308 (32.4)	2743 (34.2)	
Major cities	591 (14.4)	3361 (11.6)		583 (14.4)	1043 (13)	
SEIFA quintiles (%)			0.041			−0.002
1 (least advantaged)	878 (21.5)	6214 (21.4)		872 (21.6)	1758 (21.9)	
2	995 (24.3)	6625 (22.8)		991 (24.5)	1998 (24.9)	
3	745 (18.2)	5359 (18.5)		739 (18.3)	1495 (18.6)	
4	690 (16.9)	4547 (15.7)		686 (17)	1147 (14.3)	
5 (most advantaged)	754 (18.4)	6141 (21.2)		753 (18.6)	1633 (20.3)	
General practice site (state location)			−0.055			−0.009
New South Wales/Australian Capital Territory	1740 (42.5)	12,640 (43.6)		1713 (42.4)	3369 (41.9)	
Victoria	748 (18.3)	5956 (20.5)		742 (18.4)	1655 (20.6)	
Queensland	663 (16.2)	4486 (15.5)		656 (16.2)	1222 (15.2)	
Western Australia	332 (8.1)	1992 (6.9)		328 (8.1)	583 (7.3)	
South Australia/Northern Territory	108 (2.6)	665 (2.3)		108 (2.7)	174 (2.2)	
Tasmania	501 (12.2)	3270 (11.3)		494 (12.2)	1028 (12.8)	
Number of visits to the GP in the preceding 2 years			0.019			0.007
3–5 GP visits	487 (11.9)	3043 (10.5)		479 (11.9)	874 (10.9)	
6–14 GP visits	1101 (26.9)	8235 (28.4)		1090 (27)	2284 (28.4)	
≥15 GP visits	2504 (61.2)	17,731 (61.1)		2472 (61.2)	4873 (60.7)	
Number of renally-dependent drugs prescribed (out of the 33 drugs of interest)			−0.009			−0.036
1 drug	2532 (61.9)	17,900 (61.7)		2501 (61.9)	5043 (62.8)	
2 drugs	1026 (25.1)	7570 (26.1)		1010 (25)	2068 (25.8)	
≥3 drugs	534 (13)	3539 (12.2)		530 (13.1)	920 (11.5)	
Comorbidities						
Heart failure	874 (21.4)	6959 (24)	0.063	861 (21.3)	1913 (23.8)	0.060
Hypertension	3072 (75.1)	22,290 (76.8)	0.041	3033 (75.1)	6039 (75.2)	0.003
Stroke	1055 (25.8)	5311 (18.3)	−0.181	1032 (25.5)	2030 (25.3)	−0.006
Atrial fibrillation	1268 (31)	9231 (31.8)	0.018	1253 (31)	2528 (31.5)	0.010
Atrial flutter	87 (2.1)	672 (2.3)	0.013	87 (2.2)	179 (2.2)	0.005
Anxiety	1161 (28.4)	5766 (19.9)	−0.199	1142 (28.3)	2242 (27.9)	−0.008
Arthritis	2874 (70.2)	20,483 (70.6)	0.008	2842 (70.3)	5631 (70.1)	−0.005
Asthma	711 (17.4)	5468 (18.8)	0.038	700 (17.3)	1378 (17.2)	−0.004
Diabetes	1370 (33.5)	9324 (32.1)	−0.028	1351 (33.4)	2655 (33.1)	−0.008
Deep vein thrombosis	83 (2)	657 (2.3)	0.016	81 (2)	178 (2.2)	0.015
Depression	1840 (45)	8103 (27.9)	−0.360	1802 (44.6)	3542 (44.1)	−0.010
Cancer	2064 (50.4)	14,171 (48.9)	−0.032	2031 (50.3)	3974 (49.5)	−0.015
Coronary heart disease	1336 (32.6)	9540 (32.9)	0.005	1315 (32.5)	2618 (32.6)	0.001
Chronic kidney disease	315 (7.7)	2384 (8.2)	0.019	312 (7.7)	580 (7.2)	−0.019
Chronic liver disease	35 (0.9)	233 (0.8)	−0.006	35 (0.9)	63 (0.8)	−0.009
Chronic obstructive pulmonary disease	758 (18.5)	5296 (18.3)	−0.007	745 (18.4)	1472 (18.3)	−0.003
Substance abuse	149 (3.6)	668 (2.3)	−0.079	144 (3.6)	278 (3.5)	−0.005
Osteoporosis	1499 (36.6)	10,775 (37.1)	0.010	1485 (36.7)	2928 (36.5)	−0.006
Pain	1972 (48.2)	13,779 (47.5)	−0.014	1948 (48.2)	3905 (48.6)	0.008
Schizophrenia	76 (1.9)	168 (0.6)	−0.121	65 (1.6)	93 (1.2)	−0.039
eGFR (mL/min/1.73 m^2^), median (IQR)	53 (39–65)	51 (37–64)	−0.083	53 (39–65)	52 (38–65)	−0.035
eGFR category (mL/min/1.73 m^2^)			−0.084			−0.061
≥30	3617 (88.4)	24,827 (85.6)		3571 (88.4)	6935 (86.4)	
<30	475 (11.6)	4182 (14.4)		470 (11.6)	1096 (13.6)	

* Absolute standardized differences are reported. Abbreviations: eGFR, estimated glomerular filtration rate; GP, general practitioner; IQR, interquartile range; SEIFA, socioeconomic indexes for areas.

**Table 2:** Renally-dependent medicines with potentially inappropriate prescribing in patients with dementia and their matched controls from 2011–2020.

Medicines ^¶^	Patients Prescribed N (%)		Patients with Inappropriate Use of Drug N (%)		Medicines ^¶^	Patients Prescribed N (%)		Patients with Inappropriate Use of Drug N (%)	
	**Dementia**	**Control**	**Dementia**	**Control**		**Dementia**	**Control**	**Dementia**	**Control**
**Antidiabetic Medicines**					**Psychotropic Medicines**				
Sitagliptin	8 (0.2)	30 (0.4)	<5	8 (26.7)	Paroxetine	70 (1.7)	156 (1.9)	<5	-
Alogliptin	-	<5	-	<5	Duloxetine	88 (2.2)	175 (2.2)	5 (5.7)	12 (6.8)
Saxagliptin	<5	<5	-	-	Risperidone	112 (2.8)	58 (0.7)	-	<5
Vildagliptin	<5	<5	<5	<5	**Anti-dementia medicines**				
Metformin	374 (9.2)	654 (8.1)	100 (26.7)	178 (27.2)	Memantine	35 (0.9)	20 (0.2)	-	<5
**Antihypertensive medicines**					Galantamine	29 (0.7)	47 (0.6)	-	-
Olmesartan	70 (1.7)	151 (1.9)	6 (8.6)	12 (7.9)	**Antihistamines**				
Valsartan	52 (1.3)	124 (1.5)	7 (13.5)	14 (11.3)	Famotidine	13 (0.3)	32 (0.4)	<5	13 (40.6)
Spironolactone	121 (3)	388 (4.8)	27 (22.3)	126 (32.5)	Nizatidine	36 (0.9)	72 (0.9)	10 (27.8)	35 (48.6)
Moxonidine	47 (1.2)	136 (1.7)	15 (31.9)	40 (29.4)	**Anticoagulant medicines**				
**Antibiotics**					Dabigatran	55 (1.4)	103 (1.3)	<5	11 (10.7)
Nitrofurantoin	64 (1.6)	108 (1.3)	40 (62.5)	72 (66.7)	Apixaban	197 (4.9)	371 (4.6)	8 (4.1)	29 (7.8)
**Lipid lowering medicines**					Rivaroxaban	180 (4.4)	330 (4.1)	44 (24.4)	77 (23.3)
Fenofibrate	49 (1.2)	105 (1.3)	33 (67.3)	67 (63.8)	**Neurological medicines**				
Gemfibrozil	7 (0.2)	30 (0.4)	<5	7 (23.3)	Pregabalin	346 (8.6)	886 (11)	<5	37 (4.2)
Rosuvastatin	714 (17.7)	1432 (17.8)	31 (4.3)	63 (4.4)	**Antiarrhythmics**				
**Analgesic, antipyretic and anti-inflammatory medicines**					Digoxin	443 (11)	954 (11.9)	<5	6 (0.6)
Diclofenac	71 (1.7)	157 (1.9)	<5	5 (3.2)					
Ibuprofen	55 (1.4)	94 (1.2)	<5	<5					
Indomethacin	30 (0.7)	39 (0.5)	<5	<5					
Mefenamic acid	-	<5	-	-					
Naproxen	41 (1)	102 (1.3)	<5	9 (8.8)					
Meloxicam	228 (5.6)	601 (7.5)	11 (4.8)	31 (5.1)					
Celecoxib	190 (4.7)	412 (5.1)	12 (6.3)	23 (5.6)					
Etoricoxib	<5	-	-	-					

^¶^ Combination medicines were assessed for potentially inappropriate prescribing separately. Note: cell counts less than 5 suppressed.

**Table 3:** Odds ratio for factors associated with ≥1 inappropriate prescription in older patients with dementia (unmatched group) in Australian general practice from 2011–2020 (*n* = 381).

	N	At Least One Inappropriate Prescription, n (%)	Unadjusted		Adjusted ^§^	
			OR (95% CI) ^¶^	*p* Value	OR (95% CI) ^¶^	*p* Value
Age						
<75	855	59 (6.9)	Ref		Ref	
≥75	3237	322 (9.9)	1.49 (1.12–1.99)	0.007	1.13 (0.82–1.56)	0.438
Gender						
Females	2355	210 (8.9)	Ref		Ref	
Males	1737	171 (9.8)	1.11 (0.90–1.38)	0.313	1.21 (0.95–1.54)	0.125
Index year				<0.001		0.483
2011–2012	913	66 (7.2)	Ref		Ref	
2013–2014	886	96 (10.8)	1.56 (1.12–2.16)	0.008	1.34 (0.93–1.93)	0.122
2015–2016	825	80 (9.7)	1.38 (0.98–1.94)	0.065	1.14 (0.78–1.67)	0.510
2017–2018	793	76 (9.6)	1.36 (0.96–1.92)	0.080	1.08 (0.73–1.59)	0.693
2019–2020	675	63 (9.3)	1.32 (0.92–1.89)	0.130	1.32 (0.88–1.97)	0.173
Rurality				<0.001		0.661
Major cities	591	49 (8.3)	Ref		Ref	
Outer regional/remote/very remote	2155	197 (9.1)	1.11 (0.80–1.54)	0.521	1.14 (0.76–1.71)	0.520
Inner regional	1314	132 (10)	1.23 (0.88–1.74)	0.228	1.20 (0.81–1.76)	0.363
SEIFA quintiles				<0.001		0.310
1 (least advantaged)	878	78 (8.9)	1.15 (0.81–1.63)	0.442	0.81 (0.53–1.27)	0.369
2	995	100 (10.1)	1.32 (0.94–1.84)	0.110	1.11 (0.74–1.65)	0.624
3	745	75 (10.1)	1.32 (0.92–1.88)	0.129	1.20 (0.80–1.81)	0.371
4	690	66 (9.6)	1.25 (0.86–1.80)	0.241	1.14 (0.76–1.72)	0.524
5 (most advantaged)	754	59 (7.8)	Ref		Ref	
eGFR category (mL/min/1.73 m^2^)						
≥30	3617	179 (4.9)	Ref		Ref	
<30	475	202 (42.5)	14.21 (11.22–18.00)	<0.001	15.13 (11.71–19.56)	**<0.001**
Comorbidities						
Hypertension	3072	321 (10.4)	1.87 (1.40–2.48)	<0.001	1.24 (0.91–1.70)	0.174
Stroke	1055	106 (10)	1.12 (0.89–1.42)	0.339	0.94 (0.71–1.23)	0.645
Diabetes	1370	200 (14.6)	2.40 (1.94–2.97)	<0.001	2.22 (1.74–2.83)	**<0.001**
Cancer	2064	200 (9.7)	1.09 (0.89–1.35)	0.400	0.94 (0.74–1.20)	0.624
Chronic obstructive pulmonary disease	758	67 (8.8)	0.93 (0.71–1.23)	0.620	0.80 (0.58–1.10)	0.167
Atrial fibrillation	1268	100 (7.9)	0.77 (0.61–0.98)	0.036	0.60 (0.46–0.79)	**<0.001**

^¶^ Odds ratio estimated using univariable and multivariable logistic regression with robust standard errors. ^§^ Adjusted for all variables. Abbreviations: eGFR, estimated glomerular filtration rate; GP, general practitioner; SEIFA, socioeconomic indexes for areas. Note: percentages do not total 100% owing to missing data.

## Data Availability

The MedicineInsight data is now under the custodianship of the Australian Commission on Safety and Quality in Health Care (MedicineInsight@safetyandquality.gov.au).

## Supplementary Materials

The following supporting information can be downloaded at: https://www.mdpi.com/article/10.3390/jcm14134734/s1, Table S1: List of medications which require dosage adjustment or avoidance with a reduction in renal function; Table S2: Renally-dependent medications by drug class with potentially inappropriate prescribing in patients with dementia and their matched controls with average eGFR (≥30 mL/min/1.73 m^2^, and <30 mL/min/1.73 m^2^) from 2011–2020; Figure S1: Trends in potentially inappropriate prescribing of 33 renally-dependent drugs in primary care patients with dementia and their control groups without matching; Figure S2: Trends in potentially inappropriate prescribing of 33 renally-dependent drugs based on calculated eGFR category in primary care patients with dementia and their matched controls; File S1: STROBE Statement—Checklist of items that should be included in reports of cohort studies.

## Abbreviations

AMH, Australian Medicines Handbook; CKD, chronic kidney disease; CrCl, creatinine clearance; eGFR, estimated glomerular filtration rate; GP, general practitioner; NPS, National Prescribing Service; SEIFA, socioeconomic indexes for areas.
