# Supplementary material for: Ten-Year Trend in the Potentially Inappropriate Prescribing of Renally-Dependent Medicines in Australian General Practice Patients with Dementia
Source: J Clin Med. 2025 Jul 4;14(13):4734. doi: 10.3390/jcm14134734 (PMC12251500; doi:10.3390/jcm14134734)
Supplement: Supplementary file 1 [file jcm-14-04734-s001.zip › Supplementary Figure S2.pdf]

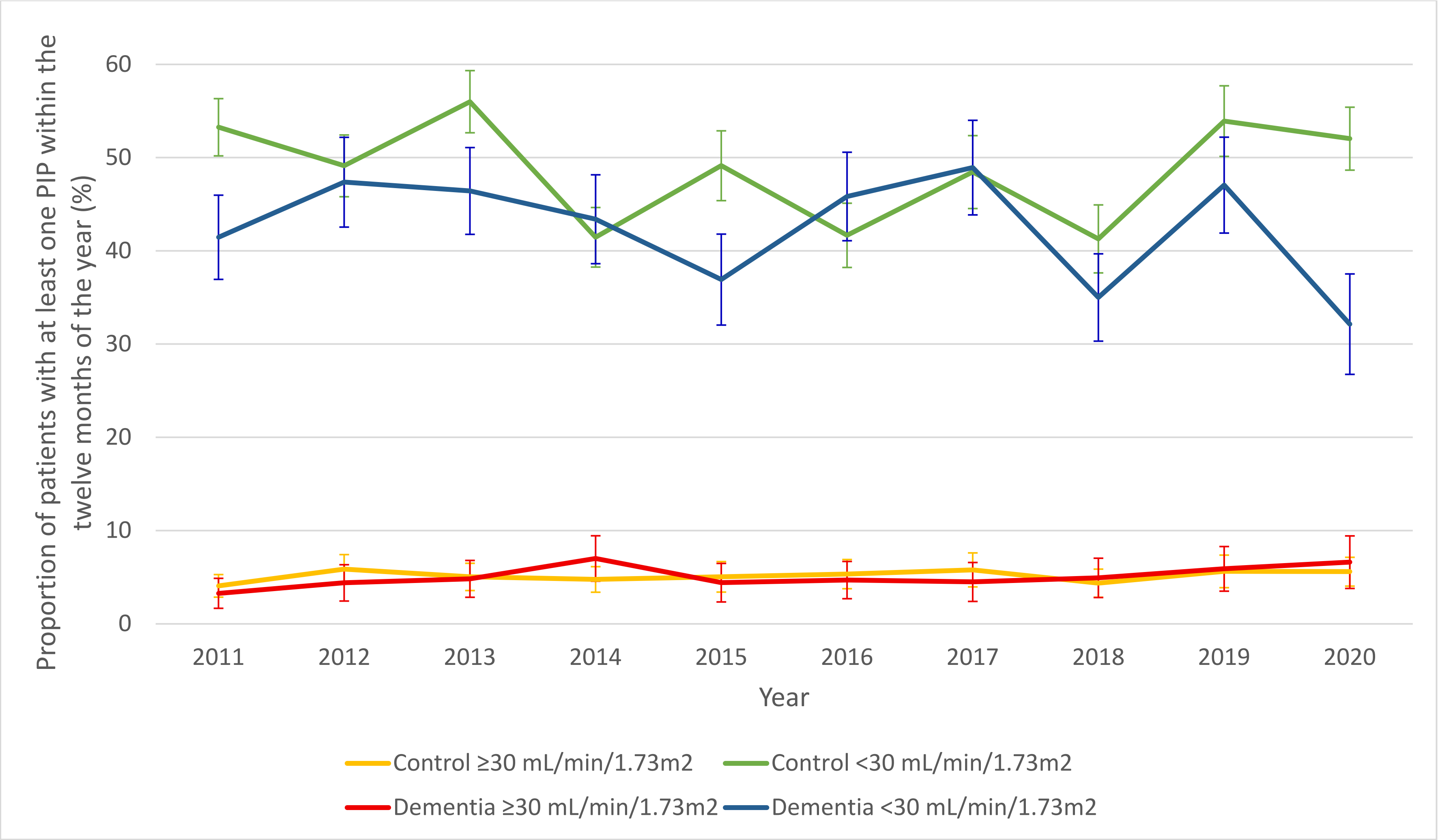


**Supplementary Figure S2.** Trends in potentially inappropriate prescribing of 33 renally-dependent drugs based on calculated eGFR category in primary care patients with dementia and their matched controls. Error bars indicate 95% confidence intervals. PIP, potentially inappropriate prescription.
