# Supplementary material for: Ten-Year Trend in the Potentially Inappropriate Prescribing of Renally-Dependent Medicines in Australian General Practice Patients with Dementia
Source: J Clin Med. 2025 Jul 4;14(13):4734. doi: 10.3390/jcm14134734 (PMC12251500; doi:10.3390/jcm14134734)
Supplement: Supplementary file 1 [file jcm-14-04734-s001.zip › Supplementary Table S1.pdf]

Supplementary Table S1. List of medications which require dosage adjustment or avoidance with a reduction in renal function.

**Medicine**	**Australian Medicines Handbook recommendations in renal impairment (maximum dose or contraindication)**
Sitagliptin	eGFR 30–45mL/min: 50mg once daily<br>eGFR <30mL/min: 25mg once daily
Alogliptin	CrCl 30–50mL/min: 12.5mg once daily<br>CrCl <30 mL/min: 6.25mg once daily
Saxagliptin	eGFR <45mL/min: 2.5mg once daily
Vildagliptin	CrCl <60 mL/min: 50mg once daily
Metformin	CrCl 60–90mL/min: 2 grams daily<br>CrCl 30–60mL/min: 1 gram daily<br>CrCl 15–30mL/min: 500mg daily<br>CrCl <15mL/min: Contraindicated
Olmesartan	CrCl <30mL/min: Contraindicated
Valsartan	CrCl <30mL/min: 80mg daily
Spironolactone	CrCl <30mL/min: Contraindicated
Moxonidine	CrCl 30–60mL/min: 200mcg daily<br>CrCl <30mL/min: Contraindicated
Nitrofurantoin	CrCl <60mL/min: Contraindicated[¶]
Fenofibrate	CrCl 30–60mL/min: 96mg once daily<br>CrCl <30mL/min: Contraindicated
Gemfibrozil	CrCl <30mL/min: Contraindicated
Diclofenac	CrCl <25mL/min: Contraindicated
Ibuprofen	CrCl <25mL/min: Contraindicated
Indomethacin	CrCl <25mL/min: Contraindicated
Mefenamic acid	CrCl <25mL/min: Contraindicated
Naproxen	CrCl <25mL/min: Contraindicated
Meloxicam	CrCl <30mL/min: Contraindicated
Celecoxib	CrCl <30mL/min: Contraindicated
Etoricoxib	CrCl <30mL/min: Contraindicated
Rosuvastatin	CrCl <30mL/min: 10mg once daily
Paroxetine	CrCl <30mL/min: 40mg once daily
Duloxetine	CrCl <30mL/min: 30mg once daily
Risperidone	CrCl <60mL/min: 4mg daily
Memantine	CrCl 5–29mL/min: 10mg once daily
Galantamine	CrCl <10mL/min: Contraindicated
Famotidine	CrCl <50mL/min: 20mg daily
Nizatidine	CrCl 20–50mL/min: 150mg once daily<br>CrCl <20mL/min: 150mg on alternate days
Dabigatran	CrCl <30mL/min: Contraindicated
Apixaban	CrCl <25mL/min: Contraindicated
Rivaroxaban	CrCl 15–50mL/min (prevention of emboli in AF): 15mg once daily<br>CrCl <15mL/min: Contraindicated
Pregabalin	CrCl 30–60mL/min: 300mg daily<br>CrCl 15–30mL/min: 150mg daily<br>CrCl <15 mL/min: 75mg daily
Digoxin	CrCl 30–60mL/min: 62.5–250mcg daily[*]<br>CrCl 10–30mL/min: 62.5–125mcg daily*

	CrCl <10mL/min: 62.5mcg once daily or on alternate days

Abbreviations: AF, atrial fibrillation; CrCl, Creatinine clearance; eGFR, estimated glomerular filtration rate.

¶ Nitrofurantoin is may be considered for short-term use (e.g. 5 days) if CrCl is 30–60 mL/min.

* Where doses were specified as a range, the maximum of the range was used in the analysis.
