# Supplementary material for: Ten-Year Trend in the Potentially Inappropriate Prescribing of Renally-Dependent Medicines in Australian General Practice Patients with Dementia
Source: J Clin Med. 2025 Jul 4;14(13):4734. doi: 10.3390/jcm14134734 (PMC12251500; doi:10.3390/jcm14134734)
Supplement: Supplementary file 1 [file jcm-14-04734-s001.zip › Supplementary Table S2.pdf]

Supplementary Table S2. Renally-dependent medications by drug class with potentially inappropriate prescribing in patients with dementia and their matched controls with average eGFR (≥30 mL/min/1.73 m^2, and <30 mL/min/1.73 m^2) from 2011–2020.

**Medication class¶**	**N (%) patients prescribed medication class**		**N (%) of patients prescribed medication class with inappropriate use**		**N (%) patients prescribed medication class**		**N (%) of patients prescribed medication class with inappropriate use**	
	**Dementia**	**Control**	**Dementia**	**Control**	**Dementia**	**Control**	**Dementia**	**Control**
	**eGFR ≥30 mL/min/1.73 m^2**				**eGFR <30 mL/min/1.73 m^2**			
**DPP-4 inhibitors**	6 (0.1)	27 (0.3)	<5	5 (18.5)	6 (0.1)	8 (0.1)	<5	5 (62.5)
**Biguanides**	344 (8.5)	592 (7.4)	79 (23)	129 (21.8)	30 (0.7)	62 (0.8)	21 (70)	49 (79)
**ARBs**	107 (2.6)	248 (3.1)	-	-	15 (0.4)	27 (0.3)	13 (86.7)	26 (96.3)
**Aldosterone antagonists**	94 (2.3)	262 (3.3)	-	-	27 (0.7)	126 (1.6)	**27 (100)**	**126 (100)**
**Alpha-2/imidazoline receptor agonists**	32 (0.8)	96 (1.2)	-	-	15 (0.4)	40 (0.5)	**15 (100)**	**40 (100)**
**Antibiotics**	58 (1.4)	93 (1.1)	34 (58.6)	57 (61.3)	6 (0.1)	15 (0.2)	**6 (100)**	**15 (100)**
**Fibrates**	41 (1)	105 (1.3)	19 (46.3)	44 (41.9)	15 (0.4)	30 (0.4)	**15 (100)**	**30 (100)**
**NSAIDs**	577 (14.3)	1318 (16.4)	-	-	39 (1)	90 (1.1)	31 (79.5)	73 (81.1)
**Statins**	653 (16.1)	1304 (16.2)	-	-	61 (1.5)	128 (1.6)	31 (50.8)	63 (49.2)
**SSRIs**	69 (1.7)	154 (1.9)	<5	-	<5	<5	-	-
**SNRIs**	77 (1.9)	157 (1.9)	-	<5	11 (0.3)	18 (0.2)	5 (45.4)	11 (61.1)
**Antipsychotics**	112 (2.8)	58 (0.7)	-	<5	-	-	-	-
**Anti-dementia drugs**	62 (1.5)	59 (0.7)	-	-	<5	8 (0.1)	-	<5
**H_2 antagonists**	41 (1)	94 (1.2)	8 (19.5)	38 (40.4)	8 (0.2)	10 (0.1)	6 (75)	**10 (100)**
**Anticoagulants**	377 (9.3)	702 (8.7)	34 (9)	62 (8.8)	55 (1.4)	102 (1.3)	21 (38.2)	55 (53.9)
**Gabapentinoids**	313 (7.7)	750 (9.3)	<5	15 (2)	33 (0.8)	136 (1.7)	<5	22 (16.2)
**Cardiac glycosides**	386 (9.5)	779 (9.7)	-	<5	57 (1.4)	175 (2.2)	<5	5 (2.8)

¶ Combination medications were assessed for potentially inappropriate prescribing separately.

Abbreviations: ARBs, angiotensin receptor blockers; DPP-4, dipeptidyl peptidase-4; NSAIDs, non-steroidal anti-inflammatory drugs; SSRIs, selective serotonin reuptake inhibitors; SNRIs, serotonin and norepinephrine reuptake inhibitors.

Note: cell counts less than 5 suppressed.
